# Surrogate Markers of Neutrophil Extracellular Trap Formation are Associated with Ischemic Outcomes and Platelet Activation after Peripheral Angioplasty and Stenting

**DOI:** 10.3390/jcm9020304

**Published:** 2020-01-22

**Authors:** Svitlana Demyanets, Stefan Stojkovic, Lisa-Marie Mauracher, Christoph W. Kopp, Johann Wojta, Johannes Thaler, Simon Panzer, Thomas Gremmel

**Affiliations:** 1 Department of Laboratory Medicine, Medical University of Vienna, Waehringer-Guertel 18-20, 1090 Vienna, Austria; svitlana.demyanets@meduniwien.ac.at; 2 Department of Internal Medicine II, Medical University of Vienna, Waehringer-Guertel 18-20, 1090 Vienna, Austria; stefan.stojkovic@meduniwien.ac.at (S.S.); christoph.kopp@meduniwien.ac.at (C.W.K.); johann.wojta@meduniwien.ac.at (J.W.); 3 Department of Internal Medicine I, Division of Hematology and Hemostaseology, Medical University of Vienna, Waehringer-Guertel 18-20, 1090 Vienna, Austria; lisa-marie.mauracher@meduniwien.ac.at (L.-M.M.); johannes.thaler@meduniwien.ac.at (J.T.); 4 Core Facilities, Medical University of Vienna, Waehringer-Guertel 18-20, 1090 Vienna, Austria; 5 Ludwig Boltzmann Institute for Cardiovascular Research, Waehringer-Guertel 18-20, 1090 Vienna, Austria; 6 Department of Blood Group Serology and Transfusion Medicine, Medical University of Vienna, Waehringer-Guertel 18-20, 1090 Vienna, Austria; simon@wsn.at; 7 Department of Internal Medicine, Cardiology and Nephrology, Landesklinikum Wiener Neustadt, Corvinusring 3-5, 2700 Wiener Neustadt, Austria

**Keywords:** peripheral artery disease, citrullinated histone H3, cell-free DNA, outcome, platelet reactivity

## Abstract

Neutrophil extracellular traps (NETs) are supposed to play a central role in atherothrombosis. We measured circulating citrullinated histone H3 (H3Cit) and cell-free DNA (cfDNA), which serve as surrogate markers of NET formation, in 79 patients with peripheral artery disease (PAD) following infrainguinal angioplasty with stent implantation. Analysis of cfDNA and H3Cit was performed using Quant-iT™ PicoGreen^®^ dsDNA Assay Kit or an ELISA, respectively. Within two years of follow-up, the primary endpoint defined as nonfatal myocardial infarction, stroke or transient ischemic attack, cardiovascular death, and >80% target vessel restenosis occurred in 34 patients (43%). Both H3Cit (HR per 1-SD: 2.72; 95% CI: 1.2–6.3; *p* = 0.019) and cfDNA (HR per 1-SD: 2.15; 95% CI: 1.1–4.2; *p* = 0.028) were associated with the primary endpoint in a univariate Cox regression analysis. Multivariate linear regression analyses showed associations between cfDNA and platelet surface expression of P-selectin (*p* = 0.006) and activated glycoprotein IIb/IIIa (*p* < 0.001) in response to arachidonic acid (AA) after adjustment for age, sex, clinical risk factors, and inflammatory markers. H3Cit was also associated with P-selectin expression in response to thrombin-receptor activating peptide (*p* = 0.048) and AA (*p* = 0.032). Circulating H3Cit and cfDNA predict ischemic outcomes after peripheral angioplasty with stent implantation, and are associated with on-treatment platelet activation in stable PAD.

## 1. Introduction

Peripheral artery disease (PAD) is a frequent manifestation of atherosclerosis that is responsible for a high rate of morbidity and mortality worldwide [1,2]. Currently, strategies to predict adverse outcomes in patients with PAD undergoing angioplasty and stenting are limited [3,4].

Neutrophil extracellular traps (NETs) are DNA- and histone-containing structures released by neutrophils, and were originally shown to fight bacteria [5]. Recently, NETs and their components were identified in atherosclerotic plaques and arterial thrombi of mice and humans [6,7,8,9,10] as well as in thrombi of patients with PAD [11].

NETs were found to play a central role in thrombosis by promoting thrombin generation [12], by activating platelets, which in turn may promote NET formation [13,14] and provide scaffolds for fibrin accumulation [15]. Moreover, NETs are able to bind von Willebrand factor (vWF) or to inactivate tissue factor pathway inhibitor (TFPI) [16] and, therefore, are capable of stimulating the coagulation cascade as well as stabilize clot formation [17,18,19]. Thrombosis goes hand in hand with inflammation during the development and progression of atherosclerosis, and NETs are involved in different aspects of the inflammatory response during this process [20,21].

Prior to NETosis, the process by which neutrophils release their interior into the extracellular space, peptidyl arginine deiminase 4 (PAD4) citrullinates arginine residues of histone H3 (H3Cit). H3Cit, together with cell-free DNA (cfDNA), serves as a surrogate marker of NET formation [22]. While H3Cit is specific for NET formation, cfDNA is a less specific NET-related biomarker. Higher levels of H3Cit were independently associated with atrial fibrillation and all-cause mortality one year after acute ischemic stroke [23]. It was shown that patients with cancer and elevated H3Cit levels have a higher risk of developing venous thromboembolism [24] and increased mortality [25,26].

However, the predictive value of circulating H3Cit and cfDNA in patients with PAD is not known. We, therefore, measured circulating H3Cit and cfDNA in PAD patients undergoing infrainguinal angioplasty with stent implantation and correlated their levels to two-year ischemic outcomes. Moreover, we correlated parameters of inflammation and platelet activation with both surrogate markers of NETs in these patients.

## 2. Patients and Methods

### 2.1. Study Population

In this prospective cohort study, 79 patients undergoing successful infrainguinal angioplasty with endovascular stent implantation were enrolled consecutively at the Division of Vascular Medicine at the Medical University of Vienna. All patients had intermittent claudication classified as Rutherford stages of PAD 2–3 due to sonographically confirmed infrainguinal artery stenosis and occlusion, respectively. All patients received long-term aspirin therapy (100 mg/day), and 75 mg of clopidogrel per day for three months following angioplasty and stenting. Clinical follow-up was assessed 1 and 2 years after the percutaneous intervention.

Exclusion criteria were a known aspirin or clopidogrel intolerance (allergic reactions, gastrointestinal bleeding), a therapy with vitamin K antagonists (warfarin, phenprocoumon, acenocoumarol) or direct oral anticoagulants (dabigatran, rivaroxaban, apixaban, edoxaban), a treatment with ticlopidine, dipyridamole or nonsteroidal anti-inflammatory drugs, a family or personal history of bleeding disorders, malignant paraproteinemias, myeloproliferative disorders or heparin-induced thrombocytopenia, severe hepatic failure, acute and chronic inflammatory diseases, known qualitative defects in thrombocyte function, a major surgical procedure within one week before enrolment, a platelet count <100,000 or >450,000/µL and a hematocrit <30% as previously described [27,28].

The study protocol was approved by the Ethics Committee of the Medical University of Vienna in accordance with the Declaration of Helsinki (Project identification code: EK 126/2007; date of approval: 20.11.2007) and written informed consent was obtained from all study participants.

### 2.2. Blood Sampling

As previously described [29], blood was drawn by aseptic venipuncture from an antecubital vein using a 21-gauge butterfly needle (0.8 × 19 mm; Greiner Bio-One, Kremsmünster, Austria) one day after the percutaneous intervention. To avoid procedural deviations, all blood samples were taken by the same physician applying a light tourniquet, which was immediately released and the samples were mixed adequately by gently inverting the tubes. After the initial 3 ml of blood had been discarded to reduce procedurally induced platelet activation, blood was drawn into a 3.8% sodium citrate Vacuette tube (Greiner Bio-One; 9 parts of whole blood, 1 part of sodium citrate 0.129 M/L) for flow cytometry [29]. To avoid investigator-related variations of the results, flow cytometry was performed by only one operator, who was blinded to clinical follow-up and parameters of NET formation.

### 2.3. Quantification of cfDNA and H3Cit

Analysis of cfDNA was performed using Quant-iT™ PicoGreen^®^ dsDNA Assay Kit (Thermo Fisher Scientific, Waltham, MA, USA) according to the manufacturer’s instructions. The assay was read on a Varioskan LUX (Thermo Fisher).

Measurement of H3Cit from plasma and preparation of H3Cit standard was performed as described in detail before [24]. Briefly, a 96 well plate was precoated with anti-histone antibody overnight at 4 °C (Cell death detection ELISA, Sigma Aldrich, St. Louis, MO, USA). After blocking with incubation buffer, standards, controls, and samples were incubated for 1.5 h at room temperature. Following washing with phosphate-buffered saline (PBS) + Tween (0.05%; Sigma Aldrich), an anti-H3Cit antibody (1:1000 ab5103; Abcam, Cambridge, MA, USA) was added and incubated for 1.5 h. After washing, anti-rabbit horseradish peroxidase- (HRP) conjugated antibody was applied for 1 h (1:5000; goat- anti-rabbit IgG HRP; Bio Rad, Laboratories, Hercules, CA, USA). After washing, incubation with TMB (3,3′,5,5′-tetramethylbenzidine; Sigma Aldrich) for 25 min resulted in a colorimetric change. The reaction was stopped using 2% sulfuric acid. The plate was read at 450 nM at on a Varioskan LUX (Thermo Fisher).

### 2.4. Determination of Platelet Surface Expression of P-selectin and Activated Glycoprotein (GP) IIb/IIIa

**Figure 1:**
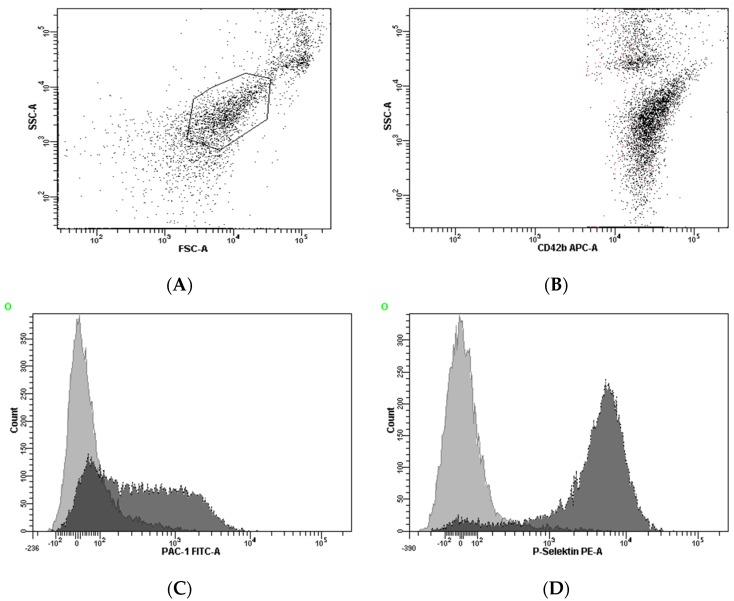
Gating strategy for the identification of the platelet population and the assessment of platelet activation. The platelet population was identified by its characteristics in the forward scatter versus side scatter plot (**A**). For analyses, this population was further identified by plotting CD42b versus side scatter (**B**). Binding of the antibodies against activated GPIIb/IIIa and P-selectin was determined in histograms for PAC-1 and P-selectin, respectively (**C**,**D**).

The expression of P-selectin and the binding of the monoclonal antibody PAC-1 to activated GPIIb/IIIa were determined in citrate-anticoagulated blood, as previously published [30,31]. In brief, whole blood was diluted in PBS to obtain 20 × 10^3^/µL platelets in 20 µL and incubated for 10 min after in vitro exposure to arachidonic acid (AA; 10 µL; final concentration 80 µM; Diamed, Cressier, Switzerland) or thrombin receptor-activating peptide (TRAP; 10 µL; final concentration 5.7 μM; Bachem, Bubendorf, Switzerland). The platelet population was identified by staining with anti-CD42b (5 µL of clone HIP1, allophycocyanin labeled, final dilution 1:9; Becton Dickinson (BD), San Jose, CA, USA), and the expression of P-selectin and activated GPIIb/IIIa were determined by the binding of the monoclonal antibodies anti-CD62p-phycoerythrin (5 µL of clone CLB-Thromb6, final dilution 1:9; Immunotech, Beckman Coulter, Fullerton, CA, USA) and PAC-1-fluorescein (5 µL, final dilution 1:9; BD), respectively. Isotype-matched control antibodies were used in separate vials for the determination of non-specific binding. After 15 min of incubation in the dark, the reaction was stopped by adding 500 µL PBS and samples were acquired immediately on a FACSCalibur flow cytometer (BD) with excitation by an argon laser at 488 nm and a red diode laser at 635 nm at a rate of 200–600 events per second. At acquisition, the platelet population was identified by its characteristics in the forward scatter versus side scatter plot (Figure 1A). A total of 10,000 events were acquired within this gate. This population was further identified by platelets stained with the platelet-specific monoclonal antibody anti-CD42b versus side scatter (Figure 1B). Binding of the antibodies against activated GPIIb/IIIa and P-selectin was determined in histograms for PAC-1 and P-selectin, respectively (Figure 1C,D). Standard BD Calibrite beads were used for daily calibration of the cytometer.

### 2.5. Clinical Endpoints

Clinical follow-up was assessed at regular visits of the study participants to the outpatient department of the Division of Vascular Medicine at the Medical University of Vienna and via telephone calls, respectively. As in previous studies [27,28], the primary endpoint was defined as the composite of the first occurrence of any of the following events: nonfatal myocardial infarction (MI), nonfatal stroke or transient ischemic attack (TIA), cardiovascular death, and sonographically confirmed >80% target vessel restenosis or reocclusion within 2 years after peripheral angioplasty and stenting.

### 2.6. Statistical Analysis

Categorical variables are summarized as counts and percentages and are compared by the χ^2^-test or by Fisher’s exact test as appropriate. Continuous variables are expressed as median and interquartile range (IQR) and compared by the t-test or by the Mann-Whitney U test in case of non-normal distribution. Univariate and multivariable Cox proportional hazard regression models were fit to assess whether the cfDNA and H3Cit could significantly predict the dichotomous clinical outcome (without/with adverse atherothrombotic events). The Benjamini-Hochberg procedure with a false discovery rate of 0.1 was applied to adjust for multiple comparisons. Model 1 was adjusted for age and sex. Model 2 was adjusted for age, sex, and clinical risk factors for PAD: active smoking, diabetes, hypertension, coronary artery disease, cerebrovascular disease, and hyperlipidaemia. Hazard ratios (HR) are given as HR per one increase of standard deviation (HR per 1-SD). Optimal cut-off values for H3Cit and cfDNA to predict adverse outcomes were calculated using Cutoff Finder’s significance of correlation with survival variable (http://molpath.charite.de/cutoff), as previously described [32]. The optimal cut-off was defined as the point with the most significant log-rank test split. Kaplan-Meier failure plots were constructed in groups according to H3Cit and cfDNA expression above or below the cut-off value to compare the time-dependent discriminative power of circulating H3Cit and cfDNA. Univariate and multivariate linear regression models were fit to evaluate the associations of H3Cit and cfDNA with platelet activation, as well as the marginal and partial impact of the variables age, sex, active smoking, diabetes, hypertension, coronary artery disease, cerebrovascular disease, hyperlipidaemia, and hs-CRP on the H3Cit and cfDNA levels. Two-sided *p*-values of 0.05 indicated statistical significance. SPSS 22.0 (IBM Corporation, Armonk, NY, USA) and STATA version 12 (StataCorp LLC, College Station, TX, USA) were used for all statistical analyses.

## 3. Results

We analyzed plasma samples from 79 PAD patients undergoing successful infrainguinal angioplasty with endovascular stent implantation. Baseline patient characteristics are shown in Table 1. The median age in our patient cohort was 65 years (IQR: 58–71 years), 50 (63.3%) patients were male, and the median BMI was 26.8 kg/m^2^ (IQR: 24.5–29.4 kg/m^2^). The prevalence of PAD-related clinical risk factors and comorbidities was the highest for hypertension (74 patients, 93.7%) and hypercholesterolemia (73 patients, 92.4%) followed by smoking in 35 patients (44.3%), diabetes in 30 patients (38%), coronary artery disease in 28 (35.5%), and cerebrovascular disease in 22 patients (27.8%). As gender is an important predictor of outcome in peripheral artery disease, and female patients with peripheral artery disease have higher mortality rates [33], we analyzed demographic and laboratory parameters separately for males and females (Table 1). Male patients more often had coronary artery disease (*p* = 0.026) and previous MI (*p* = 0.037), and lower platelet count (*p* = 0.027) as compared to female patients. Circulating levels of cfDNA were similar in men (455.7 ng/mL, IQR 380.5–690.7 ng/mL) and women (529.4 ng/mL, IQR 457.1–731.1 ng/mL, *p* = 0.132). In contrast, levels of H3Cit were higher in women (596.9 ng/mL, IQR 353.7–886.4 ng/mL) than in men (344.7 ng/mL, IQR 156.3–862.8 ng/mL, *p* = 0.020).

Median concentrations of H3Cit and cfDNA were 398.6 ng/mL (184.4–881.9 ng/mL) and 478.9 ng/mL (405.8–702.4 ng/mL), respectively. These values tend to be higher than in an age- and sex-matched cohort of 30 healthy individuals (50% male, median age 62 years (59–64 years); median H3Cit 54 ng/mL (19–166 ng/mL); median cfDNA 288 ng/mL (258–383 ng/mL)). However, since LOT numbers of ELISA kits and antibodies were not identical, one has to be very careful when interpreting the data. Within the two years of follow-up, the primary endpoint occurred in 34 patients (43%). This includes non-fatal MI in one patient, stroke or TIA in three patients, and >80% target-vessel restenosis or re-occlusion in 30 patients.

In order to investigate the predictive value of circulating H3Cit and cfDNA for the composite primary endpoint, Cox proportional hazard regression models were applied (Table 2). Both H3Cit (HR per 1-SD: 2.72, 95% CI: 1.18–6.30, *p*= 0.019) and cfDNA (HR per 1-SD: 2.15, 95% CI: 1.09–4.25, *p* = 0.028) were significantly associated with the primary endpoint in a univariate Cox regression analysis. cfDNA remained a significant predictor of the primary endpoint after adjustment for age and gender (HR per 1-SD: 2,20, 95% CI: 1109–4355, *p* = 0.024) as well as co-morbidities and clinical risk factors such as coronary artery disease, cerebrovascular disease, diabetes, active smoking, hypertension, and hyperlipidaemia (HR per 1-SD: 2.80, 95% CI: 1.34–5.84, *p* = 0.006, Table 2). The association between circulating H3Cit and the primary endpoint remained significant after adjustment for age and gender (HR per 1-SD: 2.51, 95% CI: 1.07–5.89, *p* = 0.035), but not after further adjustment for the above-mentioned co-morbidities and clinical risk factors (HR per 1-SD: 2.12, 95% CI: 0.88–5.14, *p* = 0.095, Table 2).

**Figure 2:**
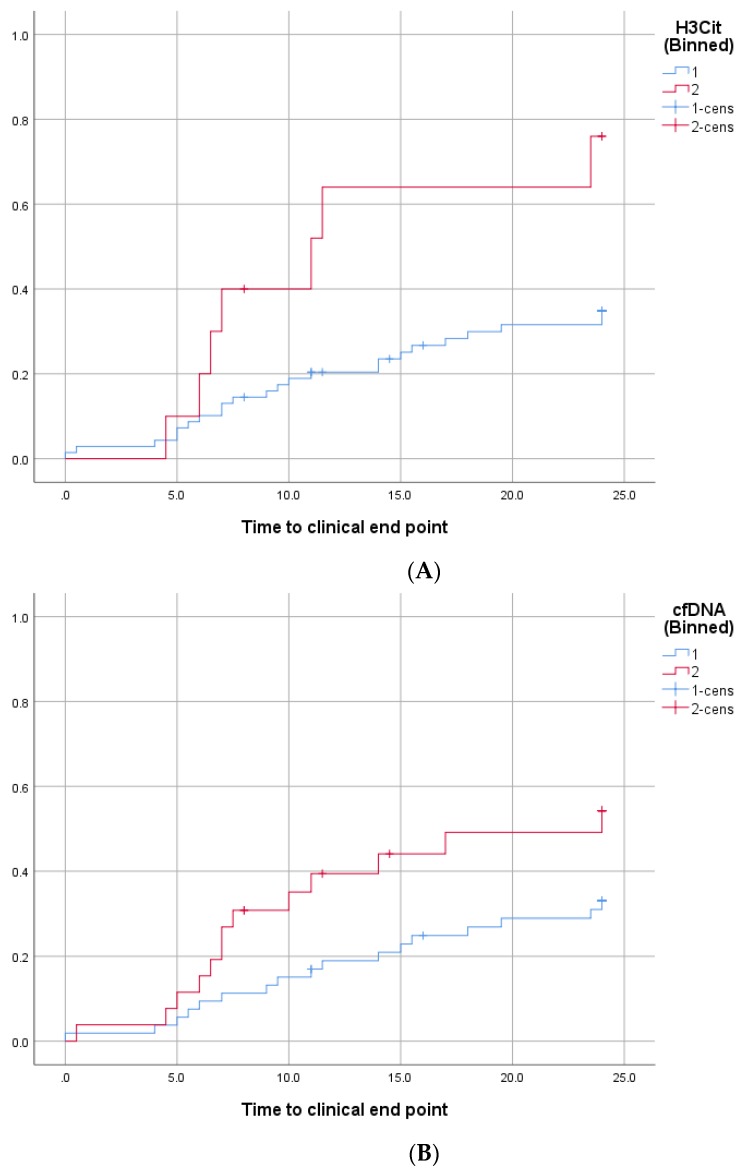
Cumulative incidence of adverse ischemic events according to circulating H3Cit (**A**) and cfDNA (**B**). Kaplan-Meier analyses for the cumulative incidence of adverse ischemic events (time to clinical endpoint) in patients with H3Cit (**A**) levels above or below the cut-off of 1128 ng/mL or cfDNA (**B**) levels above or below the cut-off of 605.9 ng/mL. The groups with circulating surrogate NET markers above the cut-off, who experienced primary endpoints, are indicated by red lines; blue lines indicate the groups with H3Cit or cfDNA levels below the cut-off.

The optimal cut-off values to predict the primary endpoint were 1128 and 605.9 ng/mL for H3Cit and cfDNA, respectively. Circulating levels of H3Cit and cfDNA above this threshold were seen in 10 (12.7%) and 26 (32.9%) patients, respectively. The primary endpoint occurred significantly more often in patients with H3Cit (log rank: *p* = 0.014) and cfDNA (log rank: *p* = 0.023) concentrations above these cut-offs than in patients with lower levels of H3Cit and cfDNA (Figure 2A,B, respectively).

Multivariate linear regression analyses showed significant associations between cfDNA and platelet surface expression of P-selectin (B = 0.033; 95% CI: 0.010–0.057; *p* = 0.006) and activated GPIIb/IIIa (B = 0.057; 95% CI: 0.029–0.086; *p* < 0.001) in response to AA after adjustment for age, sex, clinical risk factors, and inflammatory markers (Table 3). H3Cit was significantly associated with P-selectin expression in response to TRAP (B = 0.006; 95% CI: 0.001–0.012, *p* = 0.048) and AA (B = 0.065; 95% CI: 0.006–0.124; *p* = 0.032) in the same multivariate linear regression model. In the current study TRAP- and AA-inducible platelet activation were not significantly associated with the primary endpoint.

## 4. Discussion

Markers of NETs were previously detected at the site of thrombus formation and plaque rupture in different patient cohorts [8,34,35,36] including patients with PAD [11]. However, our study is the first to show a significant association between surrogate markers of NET formation and ischemic outcomes in patients with stable PAD after infrainguinal angioplasty and stenting. Both H3Cit and cfDNA were linked to the primary endpoint in univariate analyses. After adjustment for age, sex, co-morbidities, and clinical risk factors by multivariate regression analyses, only the association between cfDNA and adverse outcomes remained statistically significant. Moreover, both H3Cit and cfDNA were significantly associated with on-treatment platelet activation.

We assessed platelet surface expression of P-selectin and activated GPIIb/IIIa in response to AA and TRAP as sensitive parameters of platelet activation [37]. AA was chosen as agonist instead of adenosine diphosphate (ADP) because all patients received chronic aspirin therapy while clopidogrel was only given for three months following angioplasty and stenting. Since residual AA-inducible platelet activation reflects aspirin response, it has greater significance in the investigated cohort than ADP-inducible platelet activation. TRAP was used as a second agonist because it activates human platelets via protease-activated receptor-1, which is not inhibited by dual antiplatelet therapy with aspirin and clopidogrel. Thereby, it allows the assessment of treatment-independent platelet activation via an alternative pathway in this patient population. Moreover, we previously reported a significant association of TRAP-inducible platelet surface expression of P-selectin and activated GPIIb/IIIa with adverse ischemic outcomes following peripheral angioplasty and stenting [27]. In the current study, both TRAP- and AA-inducible platelet activation were not significantly associated with the primary endpoint. This is probably due to the smaller sample size compared to the previous study.

The biomarker potential of NET-related components has previously been shown in several clinical studies: Haumer et al. demonstrated a clear association between high neutrophil counts and future cardiovascular events in patients with symptomatic PAD [38]. Another study showed an association of plasma double-stranded DNA, as a general marker of cell death, with thrombin generation and the occurrence of arterial cardiovascular events [35]. Moreover, high levels of H3Cit were predictive for atrial fibrillation and all-cause mortality after acute ischemic stroke [23]. In cancer patients, elevated H3Cit levels may predict venous thromboembolism [24] and mortality [25,26]. However, H3Cit and cfDNA were not associated with ischemic events in cancer. This may point towards different mechanisms for developing ischemic outcomes in cancer and atherosclerosis, respectively [26]. Finally, a recent publication by Mauracher et al. demonstrated the predictive value of increased H3Cit for poor 30-day neurologic function in cardiac arrest survivors [39].

NETs possess strong prothrombotic capacities as previously shown in different experimental models. NETs induced platelet adhesion and aggregation under flow conditions, and additional incubation of human platelets with histone H3 stimulated aggregation [18]. Recombinant histone protein H3, which was purified from human neutrophils, was able to initiate the contact coagulation pathway. Nouboussie et al. observed thrombin generation in vitro in the presence of purified human neutrophil DNA in factor VII (FVII) deficient, but not in FXII deficient or FXI deficient plasma [40]. The released cfDNA is supposed to act as a milieu for the adhesion and activation of platelets and thereby as an initiating step in the process of coagulation. Additionally, histones can interact with protein C and thrombomodulin and thereby diminish the generation of activated protein C [41]. NET-related neutrophil elastase is able to degrade TFPI, and as a consequence damp the anticoagulant properties of TFPI. Moreover, the formation of a platelet-fibrin clot via platelet aggregation may be propagated by the adhesion of platelets to NETs via vWF [20]. In vitro data on the prothrombotic effects of NETs were also confirmed in animal models [17,18]. NET components may not only initiate and promote thrombosis but also influence thrombus organization and stability.

Platelet-neutrophil interaction is nowadays considered as a driver in many inflammatory and thrombotic diseases including atherosclerosis [42]. Platelets interact with neutrophils directly through neutrophil integrins and the P-selectin–P-selectin glycoprotein ligand (PSGL)-1 axis [43], or indirectly by soluble mediators such as CXC chemokines. Interestingly, platelet chemokines are able to influence NET release [44]. Furthermore, platelets promote neutrophil rolling in the sub-endothelial space via GPIb and GPIIb/IIIa. Subsequent neutrophil activation and transmigration are mediated through the binding between platelet P-selectin and neutrophil PSGL-1 [45]. P-selectin is involved in many steps of thrombus formation, e.g., by promoting platelet adhesion to the endothelium, aggregation of platelets and leukocytes, and induction of TF expression. Our findings of a significant association between platelet surface P-selectin expression and both H3Cit and cfDNA fit nicely to previously published data on augmented NET formation via P-selectin in mice [46]. Moreover, the significant association of activated GPIIb/IIIa with H3Cit and cfDNA in our study population underlines the role of platelet activation for NET formation [20]. On the other hand, histones induced P-selectin expression in gel-filtered platelets through TLR-2 and TLR-4 dependent mechanisms [47]. In summary, these observations illustrate the interdependency between platelet activation and NET formation: while increased platelet activation enhances NET formation [20]. NETs themselves foster platelet activation. The resulting vicious circle may, at least in part, explain the high risk of ischemic events in these patients. Interestingly, aspirin inhibited NET formation in stimulated human neutrophils in vitro as well as in mice in vivo [48], thereby indicating that antiplatelet therapy may be able to breach the above-described circuit. However, all of our patients received dual antiplatelet therapy with aspirin and clopidogrel for three months followed by life-long aspirin therapy, and still, both surrogate markers for NET formation were linked to ischemic outcomes. Since H3Cit and cfDNA were independently associated with AA-inducible expression of P-selectin and activated GPIIb/IIIa in the investigated cohort, one may speculate that in particular, patients with an attenuated aspirin response, are prone to NET formation.

A limitation of the implementation of H3Cit measurements in clinical routine is the lack of standardization. However, we used an ELISA for the measurement of H3Cit that has been applied in several other clinical studies [22,24,26,39]. A limitation of our study is its relatively small sample size for both patients and healthy controls. Furthermore, the primary aim of our study was to determine the predictive value of surrogate markers of NETs in patients with PAD. Nevertheless, we additionally measured cfDNA and H3Cit in age- and sex-matched healthy individuals. These data, however, need to be interpreted with caution since LOT numbers of ELISA kits and antibodies were not identical. In the patient population, we measured all parameters in blood samples collected 24 h after the intervention. Consequently, we cannot provide preprocedural values or data on the variability of NET formation and platelet activation over time. This time point was chosen because (1) 24 h after the elective procedures, all patients were still at the inpatient ward, and (2) we sought to investigate whether or not a single postprocedural measurement may be used for risk stratification. Due to its small sample size, our study should be considered hypothesis-generating only. Finally, neutrophil counts were not available in our patient population.

## 5. Conclusions

In conclusion, circulating H3Cit and cfDNA predict ischemic outcomes after peripheral angioplasty with stent implantation, and are associated with on-treatment platelet activation in stable PAD. These findings have potential implications for prognosis and risk stratification in PAD and warrant further investigations in larger patient populations.

## Figures and Tables

**Table 1:** Patient characteristics.

	Overall (*n* = 79)	Male (*n* = 50)	Female (*n* = 29)	*p*-Value
**Demographics**				
Age	65.0 (58.0–71.0)	63.5 (56.7–70.2)	67.0 (62.5–74.5)	0.080
BMI	26.8 (24.5–29.4)	27.7 (24.9–29.4)	25.7 (23.7–29.5)	0.342
Active smoking	35 (44.3)	24 (48)	11 (37.9)	0.385
**Co-Morbidities**				
Hypercholesterolemia	73 (92.4)	45 (90)	28 (96.6)	0.289
Hypertension	74 (93.7)	47 (94)	27 (93.1)	0.875
Diabetes mellitus	30 (38)	20 (40)	10 (34.5)	0.626
CAD	28 (35.5)	23 (46)	5 (17.2)	0.026
CVD	22 (27.8)	15 (30)	7 (24.1)	0.575
Previous MI	15 (19)	13 (26)	2 (6.9)	0.037
Previous TIA/stroke	9 (11.4)	6 (12)	3 (10.3)	0.527
**Laboratory Parameters**				
Triglycerides, mg/dL	143.0 (111.0–202.0)	134.0 (106.7–182.2)	162.0 (127.5–240.0)	0.093
Cholesterol, mg/dL	170.0 (143.0–214.0)	168.5 (141.7–208.2)	186.0 (146.5–230.5)	0.246
LDL, mg/dL	96.6 (67.6–124.6)	94.0 (68.7–121.3)	107.6 (66.2–136.2)	0.615
HDL, mg/dL	46.0 (40.0–53.0)	43.5 (38.7–51.5)	47.0 (43.5–54.5)	0.056
hs-CRP, mg/dL	1.05 (1.1–1.8)	0.9 (0.3–1.6)	1.3 (0.4–1.8)	0.384
Platelet count, G/L	212.0 (186.0–250.0)	205.5 (169.0–227.2)	227.0 (199.0–275.0)	0.027
Serum creatinine, mg/dL	1.03 (0.96–1.18)	1.0 (0.9–1.1)	1.0 (0.8–1.2)	0.114
**Medication**				
Aspirin	79 (100)	50 (100)	29 (100)	1
Clopidogrel	79 (100)	50 (100)	29 (100)	1
Statin	71 (89.9)	45 (90)	26 (89.7)	0.961
ACE-inhibitors/ARB	70 (88.6)	45 (90)	25 (86.2)	0.665
Beta blockers	48 (60.8)	32 (64)	16 (55.2)	0.439
Calcium channel blockers	31 (39.2)	22 (44)	9 (31)	0.255

Continuous data are shown as median (interquartile range). Dichotomous data are shown as *n* (%). BMI, body mass index; CAD, coronary artery disease; CVD, cerebrovascular disease; MI, myocardial infarction; TIA, transient ischemic attack; hs-CRP, high sensitivity C-reactive protein; IL-6, interleukin-6; ACE, angiotensin converting enzyme; ARB, angiotensin receptor blockers.

**Table 2:** Prognostic value of circulating H3Cit and cfDNA for the primary endpoint in univariate and multivariate Cox regression analyses.

	HR per	95% CI	*p*-Value
	1-SD		
H3Cit	2.72	1.18–6.30	0.019
cfDNA	2.15	1.09–4.25	0.028
**Model 1**			
H3Cit	2.51	1.07–5.89	0.035
cfDNA	2.2	1.11–4.36	0.024
**Model 2**			
H3Cit	2.12	0.88–5.14	0.095
cfDNA	2.8	1.34–5.84	0.006

Multivariate Cox regression Model 1: adjusted for age and sex; Multivariate Cox regression Model 2: adjusted for age, sex, active smoking, diabetes, coronary artery disease, cerebrovascular disease, hypertension, and hyperlipidemia. H3Cit, citrullinated histone H3; cfDNA, cell-free DNA; HR per 1-SD, Hazard ratio per one increase of standard deviation; CI, confidence interval.

**Table 3:** Relationships of circulating H3Cit and cfDNA with the parameters of platelet reactivity in multivariate linear regression analysis.

	H3Cit			cfDNA		
	B	95% CI	*p*-Value	B	95% CI	*p*-Value
*p*-selectin AA	0.065	0.006–0.124	0.032	0.033	0.010–0.057	0.006
P-selectin TRAP	0.006	0.001–0.012	0.048	0.001	−0.001–0.002	0.332
GPIIb/IIIa AA	0.089	−0.028–0.206	0.133	0.057	0.029–0.086	<0.001
GPIIb/IIIa TRAP	0.051	−0.041–0.142	0.272	−0.006	−0.041–0.28	0.712

Mutlivariate linear regression model: adjustment for age, sex, coronary artery disease, cerebrovascular disease, active smoking, diabetes, hypertension, hyperlipidaemia, and hsCRP. hs-CRP, high sensitivity C-reactive protein.

## References

[1] Song P., Rudan D., Zhu Y., Fowkes F.J.I., Rahimi K., Fowkes F.G.R., Rudan I. (2019). Global, regional, and national prevalence and risk factors for peripheral artery disease in 2015: An updated systematic review and analysis. Lancet Glob. Health.

[2] Duff S., Mafilios M.S., Bhounsule P., Hasegawa J.T. (2019). The burden of critical limb ischemia: A review of recent literature. Vasc. Health Risk Manag..

[3] Hiramoto J.S., Teraa M., de Borst G.J., Conte M.S. (2018). Interventions for lower extremity peripheral artery disease. Nat. Rev. Cardiol..

[4] Vlachopoulos C., Xaplanteris P., Aboyans V., Brodmann M., Cifkova R., Cosentino F., De Carlo M., Gallino A., Landmesser U., Laurent S. (2015). The role of vascular biomarkers for primary and secondary prevention. A position paper from the European Society of Cardiology Working Group on peripheral circulation: Endorsed by the Association for Research into Arterial Structure and Physiology (ARTERY) Society. Atherosclerosis.

[5] Brinkmann V., Reichard U., Goosmann C., Fauler B., Uhlemann Y., Weiss D.S., Weinrauch Y., Zychlinsky A. (2004). Neutrophil extracellular traps kill bacteria. Science.

[6] Quillard T., Araujo H.A., Franck G., Shvartz E., Sukhova G., Libby P. (2015). TLR2 and neutrophils potentiate endothelial stress, apoptosis and detachment: Implications for superficial erosion. Eur. Heart J..

[7] Stakos D.A., Kambas K., Konstantinidis T., Mitroulis I., Apostolidou E., Arelaki S., Tsironidou V., Giatromanolaki A., Skendros P., Konstantinides S. (2015). Expression of functional tissue factor by neutrophil extracellular traps in culprit artery of acute myocardial infarction. Eur. Heart J..

[8] Mangold A., Alias S., Scherz T., Hofbauer T., Jakowitsch J., Panzenbock A., Simon D., Laimer D., Bangert C., Kammerlander A. (2015). Coronary neutrophil extracellular trap burden and deoxyribonuclease activity in ST-elevation acute coronary syndrome are predictors of ST-segment resolution and infarct size. Circ. Res..

[9] Pertiwi K.R., van der Wal A.C., Pabittei D.R., Mackaaij C., van Leeuwen M.B., Li X., de Boer O.J. (2018). Neutrophil Extracellular Traps Participate in All Different Types of Thrombotic and Haemorrhagic Complications of Coronary Atherosclerosis. Thromb. Haemost..

[10] Novotny J., Chandraratne S., Weinberger T., Philippi V., Stark K., Ehrlich A., Pircher J., Konrad I., Oberdieck P., Titova A. (2018). Histological comparison of arterial thrombi in mice and men and the influence of Cl-amidine on thrombus formation. PLoS ONE.

[11] Farkas A.Z., Farkas V.J., Gubucz I., Szabo L., Balint K., Tenekedjiev K., Nagy A.I., Sotonyi P., Hidi L., Nagy Z. (2019). Neutrophil extracellular traps in thrombi retrieved during interventional treatment of ischemic arterial diseases. Thromb. Res..

[12] Gould T.J., Vu T.T., Swystun L.L., Dwivedi D.J., Mai S.H., Weitz J.I., Liaw P.C. (2014). Neutrophil extracellular traps promote thrombin generation through platelet-dependent and platelet-independent mechanisms. Arter. Thromb. Vasc. Biol..

[13] Andrews R.K., Arthur J.F., Gardiner E.E. (2014). Neutrophil extracellular traps (NETs) and the role of platelets in infection. Thromb. Haemost..

[14] Carestia A., Kaufman T., Schattner M. (2016). Platelets: New Bricks in the Building of Neutrophil Extracellular Traps. Front. Immunol..

[15] Kaplan M.J., Radic M. (2012). Neutrophil extracellular traps: Double-edged swords of innate immunity. J. Immunol..

[16] Massberg S., Grahl L., von Bruehl M.L., Manukyan D., Pfeiler S., Goosmann C., Brinkmann V., Lorenz M., Bidzhekov K., Khandagale A.B. (2010). Reciprocal coupling of coagulation and innate immunity via neutrophil serine proteases. Nat. Med..

[17] Von Bruhl M.L., Stark K., Steinhart A., Chandraratne S., Konrad I., Lorenz M., Khandoga A., Tirniceriu A., Coletti R., Kollnberger M. (2012). Monocytes, neutrophils, and platelets cooperate to initiate and propagate venous thrombosis in mice in vivo. J. Expr. Med..

[18] Fuchs T.A., Brill A., Duerschmied D., Schatzberg D., Monestier M., Myers D.D., Wrobleski S.K., Wakefield T.W., Hartwig J.H., Wagner D.D. (2010). Extracellular DNA traps promote thrombosis. Proc. Natl. Acad. Sci. USA.

[19] Badimon L., Vilahur G. (2015). Neutrophil extracellular traps: A new source of tissue factor in atherothrombosis. Eur. Heart J..

[20] Van Avondt K., Maegdefessel L., Soehnlein O. (2019). Therapeutic Targeting of Neutrophil Extracellular Traps in Atherogenic Inflammation. Thromb. Haemost..

[21] Bonaventura A., Montecucco F., Dallegri F., Carbone F., Luscher T.F., Camici G.G., Liberale L. (2019). Novel findings in neutrophil biology and their impact on cardiovascular disease. Cardiovasc. Res..

[22] Thalin C., Daleskog M., Goransson S.P., Schatzberg D., Lasselin J., Laska A.C., Kallner A., Helleday T., Wallen H., Demers M. (2017). Validation of an enzyme-linked immunosorbent assay for the quantification of citrullinated histone H3 as a marker for neutrophil extracellular traps in human plasma. Immunol. Res..

[23] Valles J., Lago A., Santos M.T., Latorre A.M., Tembl J.I., Salom J.B., Nieves C., Moscardo A. (2017). Neutrophil extracellular traps are increased in patients with acute ischemic stroke: Prognostic significance. Thromb. Haemost..

[24] Mauracher L.M., Posch F., Martinod K., Grilz E., Daullary T., Hell L., Brostjan C., Zielinski C., Ay C., Wagner D.D. (2018). Citrullinated histone H3, a biomarker of neutrophil extracellular trap formation, predicts the risk of venous thromboembolism in cancer patients. J. Thromb. Haemost..

[25] Thalin C., Lundstrom S., Seignez C., Daleskog M., Lundstrom A., Henriksson P., Helleday T., Phillipson M., Wallen H., Demers M. (2018). Citrullinated histone H3 as a novel prognostic blood marker in patients with advanced cancer. PLoS ONE.

[26] Grilz E., Mauracher L.M., Posch F., Konigsbrugge O., Zochbauer-Muller S., Marosi C., Lang I., Pabinger I., Ay C. (2019). Citrullinated histone H3, a biomarker for neutrophil extracellular trap formation, predicts the risk of mortality in patients with cancer. Br. J. Haematol..

[27] Gremmel T., Steiner S., Seidinger D., Koppensteiner R., Panzer S., Kopp C.W. (2014). In vivo and protease-activated receptor-1-mediated platelet activation but not response to antiplatelet therapy predict two-year outcomes after peripheral angioplasty with stent implantation. Thromb. Haemost..

[28] Gremmel T., Wadowski P.P., Mueller M., Kopp C.W., Koppensteiner R., Panzer S. (2016). Serum Cholinesterase Levels Are Associated With 2-Year Ischemic Outcomes After Angioplasty and Stenting for Peripheral Artery Disease. J. Endovasc. Ther..

[29] Gremmel T., Steiner S., Seidinger D., Koppensteiner R., Panzer S., Kopp C.W. (2009). Comparison of methods to evaluate clopidogrel-mediated platelet inhibition after percutaneous intervention with stent implantation. Thromb. Haemost..

[30] Gremmel T., Koppensteiner R., Panzer S. (2015). Comparison of Aggregometry with Flow Cytometry for the Assessment of Agonists -Induced Platelet Reactivity in Patients on Dual Antiplatelet Therapy. PLoS ONE.

[31] Gremmel T., Xhelili E., Steiner S., Koppensteiner R., Kopp C.W., Panzer S. (2014). Response to antiplatelet therapy and platelet reactivity to thrombin receptor activating peptide-6 in cardiovascular interventions: Differences between peripheral and coronary angioplasty. Atherosclerosis.

[32] Budczies J., Klauschen F., Sinn B.V., Gyorffy B., Schmitt W.D., Darb-Esfahani S., Denkert C. (2012). Cutoff Finder: A comprehensive and straightforward Web application enabling rapid biomarker cutoff optimization. PLoS ONE.

[33] Olinic D.M., Spinu M., Olinic M., Homorodean C., Tataru D.A., Liew A., Schernthaner G.H., Stanek A., Fowkes G., Catalano M. (2018). Epidemiology of peripheral artery disease in Europe: VAS Educational Paper. Int. Angiol..

[34] de Boer O.J., Li X., Teeling P., Mackaay C., Ploegmakers H.J., van der Loos C.M., Daemen M.J., de Winter R.J., van der Wal A.C. (2013). Neutrophils, neutrophil extracellular traps and interleukin-17 associate with the organisation of thrombi in acute myocardial infarction. Thromb. Haemost..

[35] Borissoff J.I., Joosen I.A., Versteylen M.O., Brill A., Fuchs T.A., Savchenko A.S., Gallant M., Martinod K., Ten Cate H., Hofstra L. (2013). Elevated levels of circulating DNA and chromatin are independently associated with severe coronary atherosclerosis and a prothrombotic state. Arterioscler. Thromb. Vasc. Biol..

[36] Riegger J., Byrne R.A., Joner M., Chandraratne S., Gershlick A.H., Ten Berg J.M., Adriaenssens T., Guagliumi G., Godschalk T.C., Neumann F.J. (2016). Histopathological evaluation of thrombus in patients presenting with stent thrombosis. A multicenter European study: A report of the prevention of late stent thrombosis by an interdisciplinary global European effort consortium. Eur. Heart J..

[37] Gremmel T., Frelinger A.L., Michelson A.D. (2016). Platelet Physiology. Semin. Thromb. Hemost..

[38] Haumer M., Amighi J., Exner M., Mlekusch W., Sabeti S., Schlager O., Schwarzinger I., Wagner O., Minar E., Schillinger M. (2005). Association of neutrophils and future cardiovascular events in patients with peripheral artery disease. J. Vasc. Surg..

[39] Mauracher L.M., Buchtele N., Schorgenhofer C., Weiser C., Herkner H., Merrelaar A., Spiel A.O., Hell L., Ay C., Pabinger I. (2019). Increased Citrullinated Histone H3 Levels in the Early Post-Resuscitative Period Are Associated with Poor Neurologic Function in Cardiac Arrest Survivors-A Prospective Observational Study. J. Clin. Med..

[40] Noubouossie D.F., Whelihan M.F., Yu Y.B., Sparkenbaugh E., Pawlinski R., Monroe D.M., Key N.S. (2017). In vitro activation of coagulation by human neutrophil DNA and histone proteins but not neutrophil extracellular traps. Blood.

[41] Ammollo C.T., Semeraro F., Xu J., Esmon N.L., Esmon C.T. (2011). Extracellular histones increase plasma thrombin generation by impairing thrombomodulin-dependent protein C activation. J. Thromb. Haemost..

[42] Soehnlein O. (2018). Decision shaping neutrophil-platelet interplay in inflammation: From physiology to intervention. Eur. J. Clin. Investig..

[43] Gremmel T., Koppensteiner R., Kaider A., Eichelberger B., Mannhalter C., Panzer S. (2015). Impact of variables of the P-selectin—P-selectin glycoprotein ligand-1 axis on leukocyte-platelet interactions in cardiovascular disease. Thromb. Haemost..

[44] Rossaint J., Herter J.M., Van Aken H., Napirei M., Doring Y., Weber C., Soehnlein O., Zarbock A. (2014). Synchronized integrin engagement and chemokine activation is crucial in neutrophil extracellular trap-mediated sterile inflammation. Blood.

[45] Sreeramkumar V., Adrover J.M., Ballesteros I., Cuartero M.I., Rossaint J., Bilbao I., Nacher M., Pitaval C., Radovanovic I., Fukui Y. (2014). Neutrophils scan for activated platelets to initiate inflammation. Science.

[46] Etulain J., Martinod K., Wong S.L., Cifuni S.M., Schattner M., Wagner D.D. (2015). P-selectin promotes neutrophil extracellular trap formation in mice. Blood.

[47] Semeraro F., Ammollo C.T., Morrissey J.H., Dale G.L., Friese P., Esmon N.L., Esmon C.T. (2011). Extracellular histones promote thrombin generation through platelet-dependent mechanisms: Involvement of platelet TLR2 and TLR4. Blood.

[48] Lapponi M.J., Carestia A., Landoni V.I., Rivadeneyra L., Etulain J., Negrotto S., Pozner R.G., Schattner M. (2013). Regulation of neutrophil extracellular trap formation by anti-inflammatory drugs. J. Pharmacol. Expr. Ther..

